# Stearoyl CoA Desaturase-1 Silencing in Glioblastoma Cells: Phospholipid Remodeling and Cytotoxicity Enhanced upon Autophagy Inhibition

**DOI:** 10.3390/ijms232113014

**Published:** 2022-10-27

**Authors:** Catarina M. Morais, Ana M. Cardoso, Ana Rita D. Araújo, Ana Reis, Pedro Domingues, Maria Rosário M. Domingues, Maria C. Pedroso de Lima, Amália S. Jurado

**Affiliations:** 1 Department of Life Sciences, University of Coimbra, Calçada Martim de Freitas, 3000-456 Coimbra, Portugal; 2 CNC—Centre for Neuroscience and Cell Biology, CIIB—Centre for Innovative Biomedicine and Biotechnology, IIIUC—Institute for Interdisciplinary Research, University of Coimbra, 3004-504 Coimbra, Portugal; 3 Mass Spectrometry Centre, LAQV-REQUIMTE, Department of Chemistry, University of Aveiro, Santiago University Campus, 3810-193 Aveiro, Portugal; 4 CESAM—Centre for Environmental and Marine Studies, Department of Chemistry, University of Aveiro, Santiago University Campus, 3810-193 Aveiro, Portugal

**Keywords:** stearoyl CoA-desaturase 1 (SCD1), glioblastoma, RNA interference technology, mass spectrometry, fatty acid and phospholipid composition, cell viability and proliferation, mitochondria respiratory activity, autophagy inhibition

## Abstract

Modulation of lipid metabolism is a well-established cancer hallmark, and SCD1 has been recognized as a key enzyme in promoting cancer cell growth, including in glioblastoma (GBM), the deadliest brain tumor and a paradigm of cancer resistance. The central goal of this work was to identify, by MS, the phospholipidome alterations resulting from the silencing of SCD1 in human GBM cells, in order to implement an innovative therapy to fight GBM cell resistance. With this purpose, RNAi technology was employed, and low serum-containing medium was used to mimic nutrient deficiency conditions, at which SCD1 is overexpressed. Besides the expected increase in the saturated to unsaturated fatty acid ratio in SCD1 silenced-GBM cells, a striking increase in polyunsaturated chains, particularly in phosphatidylethanolamine and cardiolipin species, was noticed and tentatively correlated with an increase in autophagy (evidenced by the increase in LC3BII/I ratio). The contribution of autophagy to mitigate the impact of SCD1 silencing on GBM cell viability and growth, whose modest inhibition could be correlated with the maintenance of energetically associated mitochondria, was evidenced by using autophagy inhibitors. In conclusion, SCD1 silencing could constitute an important tool to halt GBM resistance to the available treatments, especially when coupled with a mitochondria disrupter chemotherapeutic.

## 1. Introduction

Cancer is one of the biggest modern civilization scourges, representing a leading cause of death worldwide. This non-communicable disease was responsible for almost 10 million deaths in 2020, according to data from the International Agency for Research on Cancer (IARC) [1]. Glioblastoma (GBM) is a rare malignancy, but, among brain cancers, it is the most common, aggressive and deadliest [2]. The standard therapy for GBM consists of surgical removal of the tumor mass from the patient, as extensively as possible, followed by radiotherapy and chemotherapy with temozolomide. However, patients suffering from this disease do not survive, on average, more than 12–15 months after diagnosis [2]. Due to the high capacity of GBM cells to infiltrate and invade normal brain tissues and, consequently, the difficulty in achieving a complete excision of the tumor, GBM often recurs, and due to the intratumoral cellular heterogeneity, the efficiency of a chemotherapy based on a single molecular target is very limited [2,3]. In the case of temozolomide, an additional challenge regards the intrinsic or acquired resistance of GBM cells to alkylating chemotherapeutics through DNA repair, involving, for example, the O^6^-methylguanine-DNA methyltransferase (MGMT), a better prognosis being expected with such treatment for patients showing methylation of the promoter of the MGMT gene [2].

Compelling evidence has shown significant benefits from using combined therapies to fight cancer diseases, including GBM [2,3]. A promising strategy relies on coupling chemotherapy to an intervention that renders cancer cells more susceptible to drug effects. Metabolism is a key target for such therapeutic intervention, since cancer cells are characterized by profound alterations in their metabolic profile, and cancer resistance to therapies has been largely attributed to metabolism reprogramming [4]. Although changes in energy metabolism in cancer cells, particularly with regard to the Warburg effect, have long been one of the main focuses of attention of researchers in cancer biology, only more recently has the modulation of lipid metabolism by these cells emerged as another important aspect to be investigated and a driver for the implementation of new cancer therapies [5,6]. In fact, cancer cells not only have increased needs for lipids as an energy supply and to form new membranes, especially in an early stage of tumorigenesis, characterized by intense proliferation and growth, but they also need to manipulate the lipid composition to prevent the effects of oxidative stress (e.g., lipid peroxidation) and ensure signaling for their survival in adverse conditions, as well as for their dissemination. Data obtained in different cell lines and in different contexts of cancer development showed that cancer cells, in addition to being able to perform the synthesis of fatty acids de novo, unlike most normal cells that meet their fatty acid needs by taking them up from the bloodstream, also use fatty acids transported from their environment. In fact, cancer cells differ from normal cells in the way they alternate between the uptake and de novo synthesis of fatty acids, and also in the way and extent they modify and degrade them [5]. In terms of fatty acid modifications, a crucial role is played by stearoyl CoA-desaturase 1 (SCD1), an endoplasmic reticulum (ER)-resident enzyme that catalyzes the insertion of a double bond in the *cis*-∆^9^ position of saturated fatty acids, converting stearoyl-CoA (C18) or, less extensively, palmitoyl-CoA (C16), into oleoyl-CoA (C18:1) and palmitoleoyl-CoA (C16:1), respectively. SCD1 is overexpressed in different malignancies, including GBM [7]. Therefore, palmitoleic and oleic acids are two common monounsaturated fatty acids (MUFAs) in cancer cells, which, besides being employed to synthesize new membrane-forming phospholipids, are preferentially incorporated in triacylglycerols and cholesteryl esters that form lipid droplets (LDs), and channel saturated fatty acids in excess to these organelles [6]. As is well known, when accumulated in membranes, saturated fatty acids constitute critical agents of lipotoxicity, with mitochondria dysfunction and ER stress being among their most deleterious effects [7,8]. LDs are critically important in preventing lipotoxic cell damage by storing saturated fatty acids as neutral and unreactive biomolecules. In this context, the increased activity of SCD1 has been recognized as playing a crucial role in tumor growth, including that of GBM, and chemoresistance [7,9] and inhibition of SCD1 expression/activity, as well as impairment of LD generation, whose accumulation is a hallmark in cancers of different origins [10], including GBM [11], have emerged as promising therapeutic targets.

LDs are very dynamic organelles and able to establish tight connections with other subcellular compartments, namely the ER (from which they are generated), mitochondria, lysosomes and autophagosomes [12]. Growing evidence indicates that such interplay is exploited by cancer cells to promote tumor progression [13]. Interestingly, SCD1 activity, lipid metabolism, and LD formation, degradation and size (related to lipid storage capacity) concur to regulate the autophagic process, which plays an important role in the context of cancer, ensuring tumor cell survival in stress conditions [7,12]. In this regard, SCD1 activity, whose overexpression contributes to cancer cell resistance under stress, was shown to support autophagosome formation [7]. On the other hand, cancer cells use LDs to promote lipid homeostasis and modulate autophagy [12]. In fact, LDs are not only clients of autophagosomes, where their degradation takes place through a process called lipophagy, but they also regulate autophagy [6,10]. They provide lipid molecules as structural components for the formation of autophagosome membranes and signaling that trigger the expression of autophagy-related genes [10].

In the present work, an extensive characterization of lipid alterations (in terms of fatty acid content and phospholipid classes and species) of GBM cells upon SCD1 silencing was carried out, and the resulting effects on cell viability and autophagy were studied. Overall, our data support that SCD1 downregulation and autophagy impairment may concur to halt the progression of GBM, opening windows for new multimodal therapeutic approaches towards the improvement of the strategies currently in clinical use to fight this aggressive tumor.

## 2. Results

### 2.1. The Reduction of Serum Concentration in the Culture Medium of U87 Cells Promoted an Increase in the Cell-Doubling Time and in the Expression of SCD1, AMPK and LC3B II Proteins

**Figure 1:**
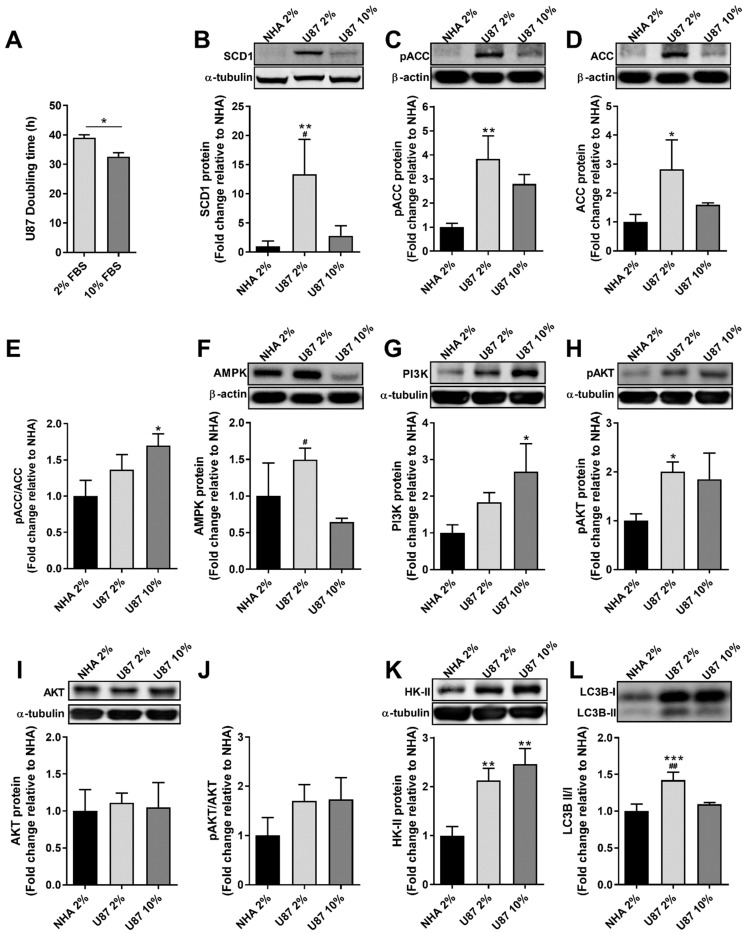
Effect of growth medium serum content on U87 cell proliferation and expression of proteins involved in cell metabolism and autophagy. Cells were plated in growth medium containing serum at a standard (10% FBS) or reduced concentration (2% FBS), and cell-doubling times were determined using the SRB assay (**A**); *n* = 2; unpaired two-sided *t*-test; * *p* < 0.05). The levels of SCD1 (**B**), pACC (**C**), ACC (**D**), pACC/ACC (**E**), AMPK (**F**), PI3K (**G**), pAKT (**H**), AKT (**I**), pAKT/AKT (**J**), HK-II (**K**) and LC3B II/I (**L**) were evaluated by Western blot analysis in normal human astrocytes (NHAs) and U87 cells at 96 h after plating. U87 cells were cultured in growth medium containing 2% or 10% FBS. Results of protein expression and protein ratio are presented with respect to NHAs (*n* = 2–3; one-way ANOVA followed by Tukey’s test; * *p* < 0.05, ** *p* < 0.01, *** *p* < 0.001 vs. NHAs; ^#^ *p* < 0.05, ^##^ *p* < 0.01 vs. U87 10% FBS).

The influence of the growth medium serum content on the proliferation rate of cancer cells has been widely reported, and GBM cells are not an exception. As shown in Figure 1A, U87 cells grown in standard serum conditions (10% FBS) presented a doubling time of ca. 33 h, in agreement with previous reports [14,15], but the reduction of the serum concentration to 2% increased the cell-doubling time by 6 h. Under these rate-limiting conditions, the expression of SCD1 protein in U87 cells increased significantly (Figure 1B), as compared to that in cells grown in 10% FBS and in normal human astrocytes (NHAs). It should be noted that NHAs are commonly cultured under low serum [16], conditions that were also employed herein, by supplementing the culture medium with 2% FBS [17,18]. However, expression of the enzyme acetyl-CoA carboxylase (ACC), which is upstream of SCD1 in the fatty acid synthesis pathway, was only slightly affected. In fact, although the levels of the metabolic inactive form (the phosphorylated ACC, pACC), in parallel with those of the total ACC, were shown to be higher in U87 cells grown in 2% FBS as compared to cells grown in 10% FBS and to NHAs; such alterations were only significant in comparison with NHAs (Figure 1C,D). Similarly, the inactivated/total ACC ratio showed no significant alterations between cells cultured in 2% and 10% FBS-containing media (Figure 1E). Expression of the AMP-activated protein kinase (AMPK), a known regulator of cellular growth and metabolism [19], significantly increased in cells grown in 2% FBS-containing medium relative to 10% FBS. The PI3K/Akt pathway, which also plays a critical role in cellular metabolism [20], did not appear to be affected by serum concentration, as no significant alterations were observed in the protein levels of PI3K (Figure 1G), activated AKT (pAKT; Figure 1H) and total AKT (Figure 1I), as well as in the activated/total AKT ratio (Figure 1J), between cells grown in the presence of 2% and 10% serum. The enzyme hexokinase-II (HK-II), which catalyzes the first step in glucose metabolism [21], although being overexpressed in U87 cells relative to NHAs, was also not affected by serum concentration (Figure 1L). However, the reduction of serum concentration promoted an increase in LC3B-II in U87 cells without changing LC3B total levels, thus increasing the LC3B II/I ratio (Figure 1L) and suggesting increased autophagy [22].

### 2.2. SCD1 Silencing Induced Toxic Effects on U87 Cells Grown in Low Serum-Containing Medium, Which Were Aggravated in the Presence of Autophagy Inhibitors

**Figure 2:**
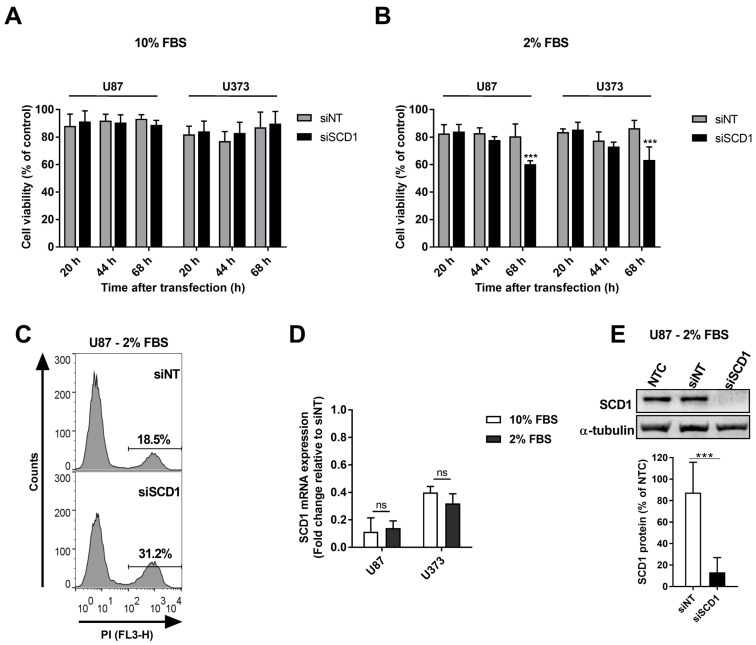
Cytotoxicity and SCD1 mRNA levels in U87 and U373 cells after SCD1 silencing. Cells grown in medium containing a standard serum concentration (10% FBS; (**A**,**D**)) or a reduced serum concentration (2% FBS; (**B**–**E**) were transfected with an siRNA against SCD1 (siSCD1) or a non-targeting siRNA sequence (siNT), complexed with Lipofectamine RNAiMAX. Viability of U87 and U373 cells (**A**,**B**) was determined 20, 44 and 68 h after transfection by the Alamar blue assay, and is expressed as the percentage of the viability of the non-treated cells (control), taken as 100% (*n* = 4–8; two-way ANOVA followed by Sidak’s test; *** *p* < 0.001 vs. siNT). U87 cell death (**C**) was assessed through PI staining, by flow cytometry, 68 h after transfection of the cells, grown in 2% serum-containing medium, with siSCD1/Lipofectamine RNAiMAX complexes. SCD1 mRNA levels (**D**) were determined by qRT-PCR in U87 and U373 cells grown in medium supplemented with 2% or 10% FBS 68 h after transfection with siSCD1 or siNT. SCD1 mRNA levels in cells transfected with siSCD1 were normalized to those expressed in cells transfected with siNT (n = 3; unpaired two-sided *t*-test; ns, not significant). SCD1 protein levels (**E**) were evaluated by Western blot analysis in U87 cells grown in 2% serum-containing medium 68 h after transfection with siSCD1 or siNT and normalized to non-treated cells (NTC) (*n* = 6; unpaired two-sided *t*-test; *** *p* < 0.001 vs. siNT).

Cell treatment with siRNAs targeting SCD1 expression (siSCD1) complexed with Lipofectamine RNAiMAX had essentially no effect on the viability of U87 and U373 human glioblastoma cells when cultured in regular growth medium, which contains 10% FBS (Figure 2A). However, when the serum concentration in the cell growth medium was decreased to 2%, a reduction of ca. 40% in the viability of U87 and U373 cells was observed 68 h after transfection with respect to non-treated cells (Figure 2B). This effect on the cell viability was significantly different from that obtained using a non-targeting siRNA sequence (siNT) as control. Under these growth-limiting conditions, siSCD1 treatment induced an increase in propidium iodide (PI)-positive U87 cells by ca. 1.7-fold, as compared to siNT-treated cells (Figure 2C). Notably, SCD1 mRNA levels in both U87 and U373 cells determined 68 h after transfection were drastically suppressed (Figure 2D), with a decay by ca. 85% and 65% in U87 and U373 cells, respectively, regardless of the content of serum in the growth medium, thus confirming the efficiency of SCD1 silencing in both cell types and in both growth conditions, i.e., in the presence of 2% and 10% serum. Consistently, siSCD1-transfected U87 cells in 2% serum-containing medium showed a pronounced decay of SCD1 protein content (by ca. 87%), with respect to non-treated cells or cells transfected with siNT (Figure 2E).

**Figure 3:**
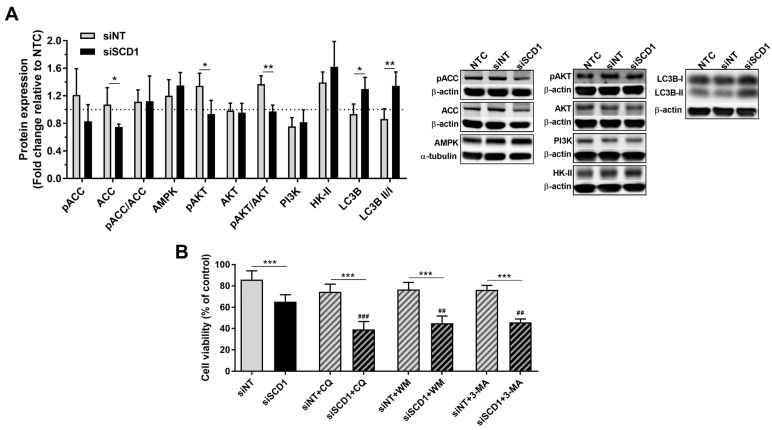
Effect of SCD1 silencing on the expression of proteins involved in the metabolism and autophagy of U87 cells and on cell viability in the presence of autophagy inhibitors. Cells grown in medium containing 2% FBS were transfected with an siRNA against SCD1 (siSCD1) or a non-targeting siRNA sequence (siNT), complexed with Lipofectamine RNAiMAX. (**A**) Sixty-eight hours after transfection, the levels of SCD1, pACC, ACC, pAKT, AKT, PI3K, HK-II and LC3B were evaluated by Western blot analysis and normalized to non-treated cells (NTC) (*n* = 3–6; unpaired two-sided t-test; * *p* < 0.05, ** *p* < 0.01, *** *p* < 0.001); the pACC/ACC and pAKT/AKT ratios were determined from the corresponding levels of the pairs pACC and ACC, and pAKT and AKT, respectively. The LC3B II/I ratio was determined from the corresponding expression levels of LC3B II and LCB3I. (**B**) Twenty hours after transfection, chloroquine (CQ), wortmannin (WM) or 3-methyladenine (3-MA) were added to U87 cells. Cell viability was evaluated 48 h later by the Alamar blue assay. Results are expressed as the percentage of the viability of NTC (*n* = 4–6; one-way ANOVA followed by Tukey’s test; *** *p* < 0.001 vs. the respective siNT; ^##^ *p* < 0.01, ^###^ *p* < 0.001 vs. siSCD1 per se; siNT per se vs. siNT + autophagy inhibitors was not significant).

Although no significant differences were observed in the levels of AMPK, PI3K and HK-II protein expression and the ratios pACC/ACC and pAKT/AKT were not significantly altered upon SCD1 silencing, as compared to control transfection or non-treated cells, the LC3B II/I ratio, in contrast, significantly increased in SCD1-silenced cells, which can be assigned to an increase in LC3B-II (leading to an increased total LC3B level), as LC3B-I levels remained constant (Figure 3A).

To address the potential contribution of autophagy in mitigating the impact of SCD1 silencing on cell viability, U87 cells were treated with classic autophagy inhibitors. Addition of chloroquine (CQ), which targets the late phase of autophagolysosomal proteolysis [23], further decreased the viability of U87 cells transfected with siSCD1 (ca. 60% of non-viable cells; Figure 3B). The same effect was observed by targeting the early phase of phagophore formation [23] with wortmannin (WM) or 3-methyladenine (3-MA), with a decrease in viable cells by ca. 55%. As shown in Figure 3B, all of the autophagy inhibitors promoted a statistically significant reduction in the viability of SCD1-silenced cells, as compared to that induced by SCD1 silencing per se.

### 2.3. SCD1 Silencing Promoted a Decrease in the Ratio of Total Monounsaturated to Saturated Fatty Acid Components of U87 Cell Lipids

The effect of SCD1 silencing on the fatty acid (FA) composition of the lipids extracted from U87 cells, grown in low serum-containing medium, was assessed by analyzing the corresponding FA methyl esters by GC-MS. Twenty different FAs were identified in non-treated U87 cells, their relative contents being identical to those of cells transfected with siNT (Table 1). The most abundant FAs were 18:1 n-9 (oleic acid; ca. 25%), 18:0 (stearic acid; ca. 15%), 16:0 (palmitic acid; ca. 19%) and 18:1 n-7 (cis-vaccenic acid; ca. 10%). The FAs 16:1 n-7 (palmitoleic acid), 20:4 n-6 (arachidonic acid), 22:5 n-3 (eicosapentaenoic acid, EPA) and 22:6 n-3 (docosahexaenoic acid, DHA) were found in the concentration range from 4 to 6%. Cell transfection with siSCD1 induced a statistically significant decrease in the relative proportions of the monounsaturated FAs (MUFAs) 16:1 n-7, 18:1 n-7, 18:1 n-9 and 20:1 FA, whereas the levels of the saturated FAs (SFAs) 18:0 and 20:0 and the polyunsaturated FAs (PUFAs) 18:2 n-6, 20:3 n-6 and 20:4 n-6 significantly increased (Table 1). Considering the total amounts of SFAs, MUFAs or PUFAs, it was observed that SCD1 silencing induced a decrease in the relative content of MUFAs accompanied by an increase in that of SFAs and PUFAs (Table 1). The identified n-6 and n-3 PUFAs equally increased upon SCD1 silencing, the n-6-to-n-3 PUFA ratio being unaltered (Table 1). Despite the increase in the relative abundance of PUFAs, the relative proportion of the total unsaturated FAs (UFAs, corresponding to MUFAs + PUFAs) decreased, which resulted in a decrease in UFA-to-SFA ratio, mainly attributed to changes in MUFA and SFA contents (Table 1).

The saturated FAs 16:0 and 18:0 are the preferred substrates of SCD1, being converted into 16:1 n-7 and 18:1 n-9 FAs, respectively (Figure S1). Using product-to-substrate ratios to estimate the activity of SCD1, a decrease in the enzyme activity for both substrates was noticed, as expected, after cell transfection with siSCD1, as indicated by the desaturation indexes SCD1-16 and SCD1-18, taken as the ratios of 16:1 n-7/16:0 and 18:1 n-9/18:0, respectively (Table 2). Using the same rationale, the activity of other enzymes involved in the biosynthesis of SFAs and MUFAs was estimated (Table 2; Figure S1). Thus, the activity of the elongase of very long-chain fatty acid (ELOVL)-6, estimated on the basis of the 18:0-to-16:0 ratio [24,25], was shown to increase upon SCD1 silencing. However, considering the ratio of 18:0 plus 18:1 n-9 to 16:0 to evaluate the elongation of 16:0, which depends on ELOVL-6 activity [25], no change was observed upon SCD1 silencing (ELOVL-6 + SCD1-18 index). Further elongation of 18:0 to 20:0 (ELOVL-1/3/7 index; [26]) was not affected by SCD1 silencing. In contrast, SCD1 silencing increased the estimated elongation of 16:1 n-7 to 18:1 n-7 by ELOVL-5/6 [25], although the total content in 16:1 n-7 plus 18:1 n-7 in relation to their precursor 16:0 (SCD1-16 + ELOVL-5/6 index) decreased, as expected, since their synthesis depends, directly or in a first stage, on SCD1 activity (Figure S1).

### 2.4. SCD1 Silencing in U87 Cells Induced Remodeling of the Phospholipid Composition

**Figure 4:**
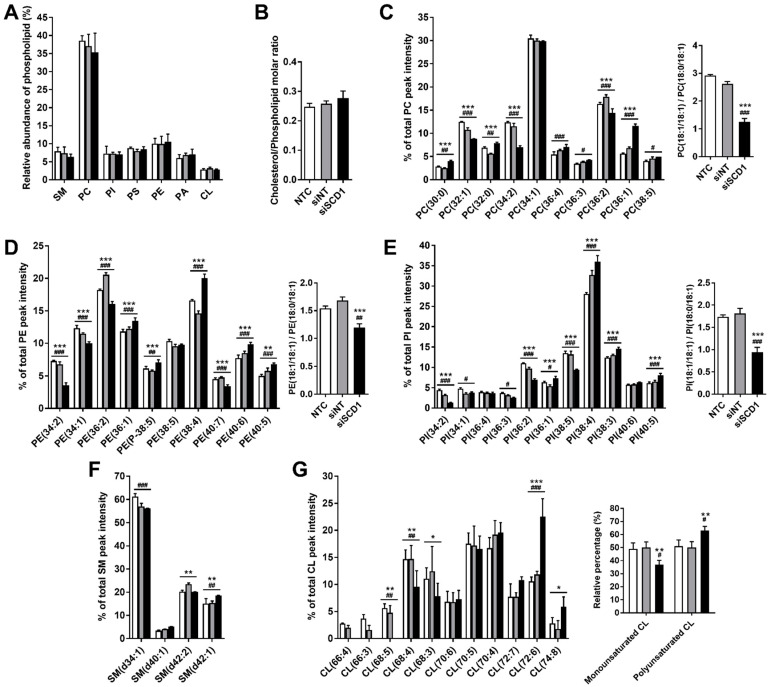
Effect of SCD1 silencing on phospholipid (PL) and cholesterol content and relative abundance of individual PL molecular species in U87 cells. Cells grown in a medium containing 2% FBS were either non-treated (NTC, white bars) or transfected with an siRNA against SCD1 (siSCD1, black bars) or a non-targeting siRNA sequence (siNT, grey bars) complexed with Lipofectamine RNAiMAX. Sixty-eight hours after transfection, lipids were extracted, and PLs analyzed by thin-layer chromatography (TLC; (**A**)) and HPLC-MS (**C**–**G**), and the cholesterol content quantified using Amplex Red Cholesterol Assay Kit (**B**). (**A**) The relative abundance of each PL class, recovered from the corresponding spot in the thin-layer chromatogram and evaluated in moles of phosphorus, is expressed as a percentage of total phospholipid in the lipid extract, estimated as the sum of phosphorus contents (in moles) presented in all of the spots of the chromatogram (*n* = 3). (**B**) The cholesterol-to-phospholipid ratios are expressed as nmol of total cholesterol per nmol of phospholipid phosphorus. (**C**–**G**) Relative abundance of the molecular species within each PL class-PC (**C**), PE (**D**), PI (**E**), SM (**F**) and CL (**G**)—analyzed by HPLC-MS in positive mode (**C**,**F**) and negative mode (**D**,**E**,**G**), is expressed as a percentage obtained from the ratio between the reconstructed area of each molecular species and the sum of all of the reconstructed areas considered for each class, multiplied by 100 (n = 2–3, two-way ANOVA followed by Tukey’s test; ^#^ *p* < 0.05, ^##^ *p* < 0.01, ^###^ *p* < 0.001 siSCD1 vs. NTC; * *p* < 0.05, ** *p* < 0.01, *** *p* < 0.001 siSCD1 vs. siNT). Species are annotated as PL(C:N), where PL is the phospholipid class, C the number of carbons, and N the number of double bonds. The ratio of the molecular species 36:2 (18:1/18:1)-to-36:1 (18:0/18:1) for the phospholipid classes PC (**C**), PE (**D**) and PI (**E**) were calculated from individual percentages.

The relative amounts of phospholipid (PL) classes in lipid extracts from U87 cells grown in low serum-containing medium and transfected with siSCD1, analyzed by TLC 68 h after transfection, showed no statistical differences as compared to those from cells treated with siNT (Figure 4A). Additionally, SCD1 silencing did not significantly alter the levels of total phospholipid in relation to cholesterol (Figure 4B).

The different PL species were then identified in order to evaluate how the changes in the FA profile (Table 1), observed upon SCD1 silencing, differentially affected the molecular composition of the various PL classes. LC-MS spectra were obtained in the positive-ion mode as [M + H]^+^ ions, in the case of phosphatidylcholine (PC) and sphingomyelin (SM), or in the negative-ion mode as [M − H]^−^ ions, in the case of phosphatidylethanolamine (PE), phosphatidylinositol (PI) and cardiolipin (CL). LC-MS/MS was performed for each ion to obtain information on fatty acyl composition. The molecular species of each phospholipid class, identified from the interpretation of the MS/MS spectra of each ion, are listed in Table S1, along with a summary of the effects promoted by SCD1 silencing.

The PC profile of U87 cells (Figure 4C) showed that the most abundant species were (in descending order) PC 34:1 (16:0/18:1), PC 36:2 (18:1/18:1), PC 32:1 (14:0/18:1 and/or 16:0/16:1), and PC 34:2 (16:0/18:2 and/or 16:1/18:1). SCD1 silencing resulted in a decrease in the relative levels of these PC species, with the exception of 34:1, which was not affected. However, in the PE profile (Figure 4D), SCD1 silencing resulted in decreased levels of the species PE 34:1 (16:0/18:1), as well as PE 34:2 (16:1/18:1) and PE 36:2 (18:1/18:1), the latter being the most abundant PE species. In the PI profile (Figure 4E), PI 36:2 (18:1/18:1) and PI 38:5 (18:1/20:4) are abundant species that also decreased after silencing of SCD1. Regarding the PL species whose relative proportions were increased upon SCD1 silencing, these included the saturated PC species PC 30:0 (14:0/16:0) and PC 32:0 (16:0/16:0), and monounsaturated PC, PE and PI species containing an oleoyl and a stearoyl chain, i.e., 36:1 (18:0/18:1). In addition, the relative content of most of the PL species combining a stearoyl chain (18:0) and a polyunsaturated acyl chain increased, including that of the most abundant PI species 38:4 (18:0/20:4). Furthermore, SCD1 silencing induced a significant decrease in the ratio of 36:2 (18:1/18:1)-to-36:1 (18:0/18:1) in all three PL classes (Figure 4C–E).

The molecular analysis of the SM class (Figure 4F) showed less dramatic changes promoted by SCD1 silencing, although a statistical difference, when compared to non-treated cells (NTC) and control transfection (siNT), was obtained for SM d42:1, which may correspond to an SM species based on a ceramide consisting of sphinganine and nervonic acid (d18:0/24:1) and/or a species based on a ceramide consisting of sphingosine and lignoceric acid (d18:1/24:0).

Regarding CL molecular composition (Figure 4G), the most abundant species in NTC and in cells treated with siNT were, in descending order, CL 70:4, CL 70:5 and CL 68:4. SCD1 silencing promoted a decrease in the relative abundance of CL 68:4 and a very significant increase in that of CL 72:6, which became the most abundant CL species upon SCD1 downregulation. An increase in the relative content of CL 74:8 was also noticed in these conditions. In addition, the CL molecular species 66:3, 66:4 and 68:5 were depleted upon SCD1 silencing. Finally, considering the sums of all monounsaturated and all polyunsaturated CL species (Figure 4G), it was observed that the silencing of SCD1 induced increased levels of polyunsaturated CL species at the expense of those of the monounsaturated ones.

**Figure 5:**
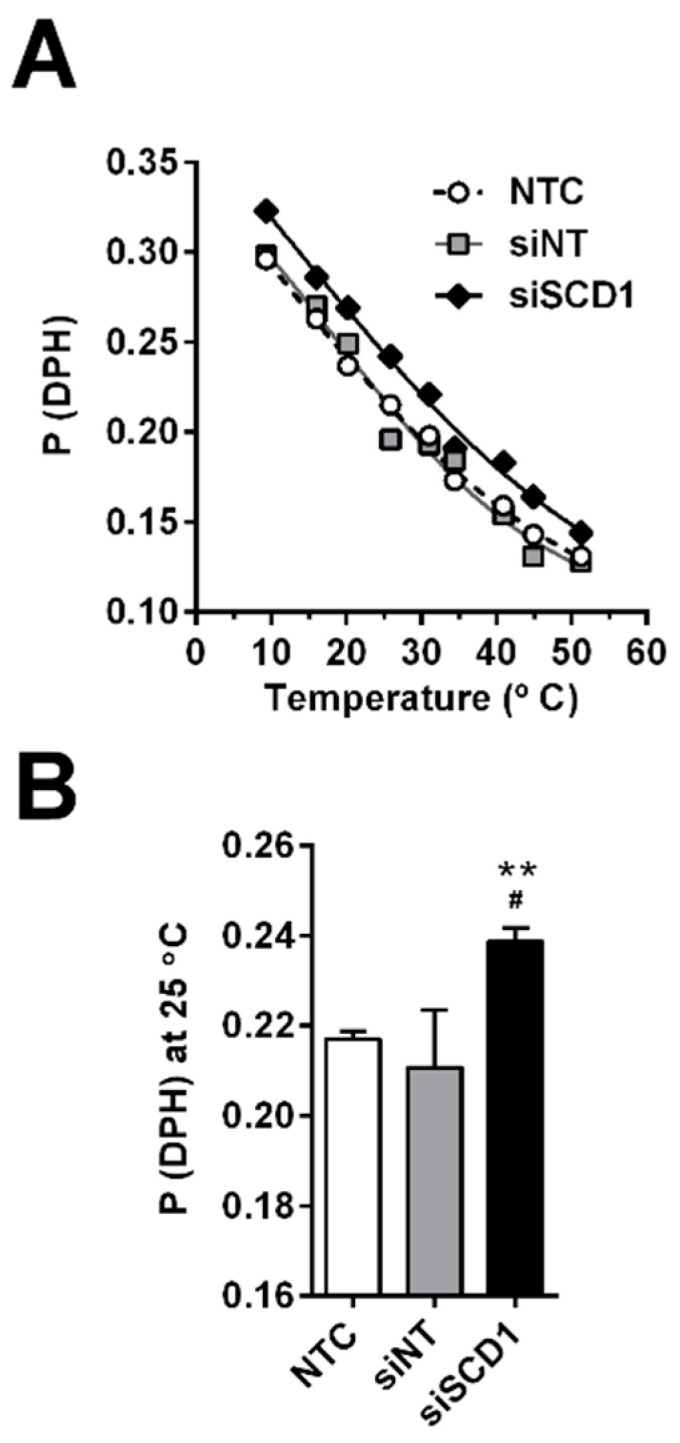
Fluorescence polarization of DPH incorporated in liposomal membranes prepared from the lipid extracts of U87 cells. Cells grown in medium containing 2% FBS were transfected with an siRNA against SCD1 (siSCD1) or a non-targeting siRNA sequence (siNT) complexed with Lipofectamine RNAiMAX. Sixty-eight hours after transfection, lipids were extracted and used to prepare liposomes, to which a small aliquot of a 2 mM DPH solution in tetrahydrofuran was added under vortex. Fluorescence polarization (P) of DPH was determined in the three liposome preparations (with respect to non-treated cells, NTC, and cells transfected with siSCD1 or siNT), over the temperature range from 10 to 50 °C (**A**). DPH fluorescence polarization values at 25 °C (**B**) were taken from thermograms similar to the typical thermogram shown in panel A, for comparison in the three liposome samples (*n* = 3; one-way ANOVA followed by Tukey’s test; ^#^ *p* < 0.05, vs. NTC; ** *p* < 0.01 vs. siNT).

Taken together, the overall effects of SCD1 silencing in U87 cells led to a decrease in the abundance of PL species containing MUFAs. Consistently, this lipid composition change was translated into an increase in hydrocarbon chain conformational order, as assessed by the increase in fluorescence polarization of the DPH probe incorporated in liposomes prepared from the lipids extracted from the cells (Figure 5).

### 2.5. SCD1 Silencing Deprived Mitochondria of U87 Cells from Their Spare Respiratory Capacity without Affecting Coupling Efficiency

**Figure 6:**
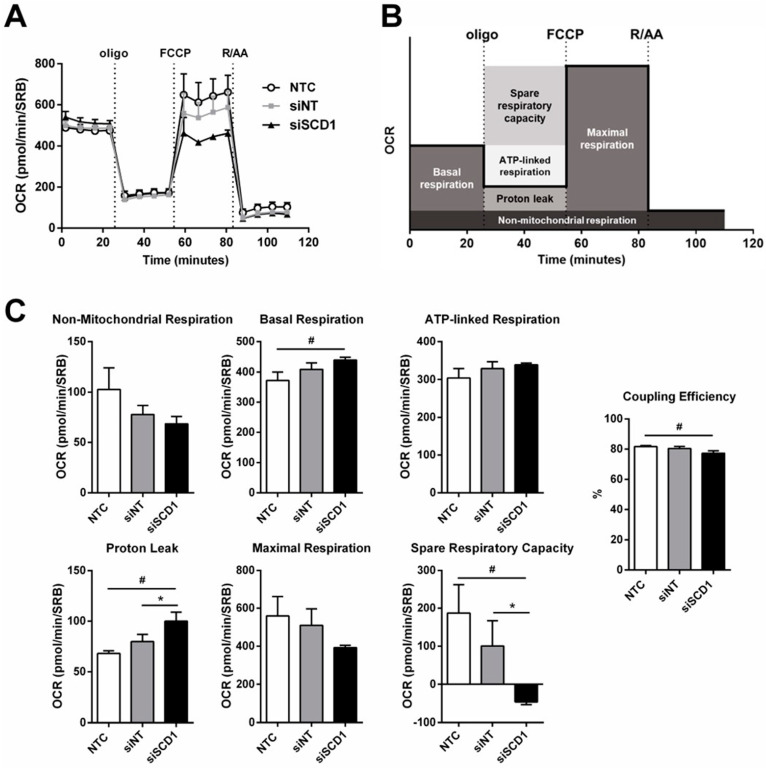
Effect of SCD1 silencing on mitochondrial respiration of U87 cells. Cells grown in medium containing 2% FBS were transfected with an siRNA against SCD1 (siSCD1) or a non-targeting siRNA sequence (siNT) complexed with Lipofectamine RNAiMAX. Sixty-eight hours after transfection, the medium was replaced with XF24 Assay Medium. Oligomycin, FCCP and rotenone plus antimycin A were diluted in XFe24 medium and loaded into the cartridge to achieve the final concentration of 1 µM for each inhibitor. Oxygen consumption rate (OCR) was recorded using a Seahorse XFe24 extracellular flux analyzer and normalized regarding the protein content of each well using the SRB assay. The real-time mitochondrial OCR profiles of non-treated cells (NTC) and cells transfected with siNT and siSCD1 after sequential injections of oligomycin (oligo), FCCP and rotenone plus antimycin A (R/AA) are shown in panel (**A**). Panel (**B**) shows how the mitochondrial respiration parameters displayed in panel (**C**) were extrapolated from an OCR profile (*n* = 3; one-way ANOVA followed by Tukey’s test; ^#^ *p* < 0.05, vs. NTC; * *p* < 0.05 versus siNT).

Since SCD1 silencing affected CL molecular composition (Figure 4G) and CL is an essential phospholipid in the regulation of mitochondrial functions, including respiratory chain activity and oxidative phosphorylation [27], the rate of oxygen consumed (OCR) by U87 cells grown in low serum-containing medium, 68 h after transfection with siSCD1 or siNT, was measured using a Seahorse XFe24 extracellular flux analyzer (Figure 6A). Values with respect to different respiratory parameters, as shown in Figure 6C, were extracted from the recorded measurements (Figure 6A), as illustrated in the Figure 6B. As shown in Figure 6C, no significant alterations were observed regarding non-mitochondrial respiration, mitochondrial basal respiration, and ATP-linked respiration between U87 cells transfected with siNT and siSCD1 (Figure 6C). The residual mitochondrial respiration that occurs by blocking the ATP synthase upon oligomycin injection, i.e., the non-ATP-linked oxygen consumption or proton leak, increased after SCD1 silencing. However, this was not enough to uncouple mitochondrial respiration, since the coupling efficiency (given by the ratio between ATP-linked respiration and mitochondrial basal respiration) was not significantly affected when compared to the control transfection. Furthermore, the maximal mitochondrial respiration, induced by uncoupling the electron transfer from oxidative phosphorylation through injection of the uncoupler FCCP, decreased after SCD1 silencing (Figure 6A,C), the cells becoming unable to achieve OCR values above the baseline, which indicates that they had lost their respiratory reserve, as it was patent in the decrease in spare respiratory capacity (Figure 6C).

## 3. Discussion

A primary requisite of cancer cells to ensure their rapid proliferation regards an enhanced supply of nucleic acids, proteins and lipids. With regard to lipid requirements, cancer cells have the capacity to switch from the uptake of fatty acids from the external medium (exogenous fatty acids) to the synthesis de novo of saturated fatty acids [5] that are converted into monounsaturated fatty acids thanks to the activity of the enzyme SCD1, which is commonly overexpressed in cancer cells [7]. The latter mechanism of providing cells with fatty acids for complex lipid synthesis is particularly suited to conditions of nutrient deficiency, a situation commonly faced by cancer cells. Cell growth in vitro depends to a large extent on the serum content of the culture medium from which the cells obtain various nutrients, including fatty acids. Thus, cancer cells cultured in the presence of low serum content are expected to become enriched in lipogenic enzymes, including SCD1, which was confirmed in the present work (as discussed below). Therefore, in the present work, aiming at evaluating the therapeutic potential of SCD1 downregulation in GBM, human U87 cells, widely used as a cellular model for GBM studies, were cultured in a medium containing 2% FBS, a serum concentration 5 times lower than that currently supplied to the growth medium classically used to culture these cells [18,28]. We should note that the growth of U87 cells was affected, although not very drastically, by the serum content in the growth medium; the cell doubling time increasing about 18% in the low serum medium as compared to that in the standard serum medium (Figure 1A). A decrease in the same order of magnitude (about 20%) was observed in the viability of U87 cells grown in 2% serum-containing medium, in the presence of an apparently non-cytotoxic oligonucleotide (siNT), as compared to cells grown in the presence of siNT in medium containing 10% serum, with no significant decay being registered under the same conditions in U373 cells (Figure 2A,B). Note that medium supplementation with FBS, the type of serum most commonly used in the culture of serum-dependent cell lines as the source of many biological molecules, including lipids [29], has been reported to promote cell growth [29,30]. Therefore, U87 cells, as well as U373 cells, should have been perturbed by the low content of serum, but somehow they must have been able to withstand the harsh conditions of the environment, possibly resorting to changes in lipid metabolism, in which SCD1 might have been involved. This hypothesis was supported by the fact that SCD1 expression significantly increased in U87 cells when the serum content decreased from 10 to 2% in the growth medium (Figure 1B). Interestingly, the expression of SCD1 was significantly lower in NHAs than in U87 cells grown in the presence of 2% serum, although not very different from that observed in U87 cells grown with 10% serum. This proves that the upregulation of SCD1 constitutes an important phenomenon that concurs with GBM cell adaptation to low nutrient conditions, as extensively reported [31]. NHAs are also poorer in the lipogenic enzyme ACC as compared to U87 cells grown in 2% serum, the content of the total ACC and of the phosphorylated (and then inactivated) form being lower in NHAs, although the pACC/ACC ratio was not statistically different from that in the U87 cells grown in those conditions. On the other hand, the decrease in serum content (from 10 to 2%) promoted a significant increase in AMPK levels (Figure 1F) and in the ratio LC3BII/I (Figure 1L) in U87 cells.

The rationale for the choice of these proteins to be analyzed in the context of the present work was as follows: ACC catalyzes a rate-limiting reaction in the biosynthesis of long-chain fatty acids, generating malonyl-CoA from acetyl-CoA, acting hence upstream of SCD1 and being indirectly regulated by this enzyme [32]. Thus, when SCD1 is downregulated, two events concur to inhibit ACC, firstly the accumulation of saturated fatty acids, which are allosteric inhibitors of ACC, secondly, the induced phosphorylation and consequent activation of AMPK (by poorly known mechanisms), which phosphorylates, and hence inactivates, ACC [7,32]. The inhibition of ACC activity, by limiting lipogenesis, constitutes an adaptation to the downregulation of SD1, thus preventing the accumulation of saturated fatty acids and their adverse effects (lipotoxicity). Accordingly, it has been demonstrated that the decrease in cancer cell growth is reinforced by ACC stimulation in SCD1-deficient cells and is reversed by addition of oleate in cells treated with an SCD1-chemical inhibitor [32]. AMPK is known as a cellular energy sensor, which, under starvation conditions, promotes cell metabolism alterations from ATP-consuming anabolic pathways as protein and fatty acid biosynthesis to ATP-producing catabolic processes as glycolysis and fatty acid oxidation, thus preserving cell energy homeostasis. On the other hand, AMPK positively regulates autophagy [7,33], which may constitute an adaptive mechanism to nutrient starvation. Therefore, the analysis of LC3B levels is also relevant in this context, the ratio LC3BII/I assuming particular importance, since LC3 lipidation (LC3B-II) is a standard indicator of autophagy [22]. It is not surprising that U87 cells grown in a medium supplied with 2% serum were enriched in AMPK and showed an increase in the ratio of LCB3-II/I, as compared with cells grown in 10% serum, the LC3BII/I ratio being also significantly lower in NHAs.

Other proteins also analyzed in U87 cells and NHAs were Akt, PI3K and HK-II, whose rationale is as follows. Akt is a serine/threonine kinase that regulates the activity and expression of multiple enzymes involved in glycolysis and fatty acid synthesis in cancer cells, thus contributing to their survival, with high levels of phosphorylated Akt being found in GBM patients with severe prognosis [34]. In order to be functionally active, Akt needs not only to be phosphorylated but also to be transferred from the cytosol to the plasma membrane, through interaction with the phosphoinositide phosphatidylinositol 3,4,5-triphosphate (PIP3). Thus, the enzyme PI3K plays a crucial role in Akt activation, because it catalyzes the conversion of phosphatidylinositol 4,5-biphosphate (PIP2) into PIP3. PI3K is stimulated by signals provided by ligands that bind tyrosine kinase receptors, such as epidermal growth factor receptor (EGFR), whose amplification and/or overexpression have been reported in about half of GBM tumors [35]. Consistently, the enzymes PI3K and pAkt involved in the signaling pathway that promotes GBM cell survival showed significantly higher levels in U87 cells than in NHAs (Figure 1G,H). HK-II, which catalyzes the rate-limiting first step of glycolysis and thus has a key role in energy and glucose-mediated lipid metabolism, is one of the multiple enzymes transcriptionally regulated by Akt [21] and is up-regulated in several types of tumors, including GBM [36,37]. Accordingly, HK-II was also more expressed in U87 cells as compared to NHAs (Figure 1K), which indicates the importance of the glycolytic metabolism for GBM cells. On the other hand, HK-II has been reported to promote autophagy in glucose-deficient conditions, such process being mediated by HK-II binding to mTORC1 (mammalian target of rapamycin complex 1), resulting in a decrease in the autophagy-depressing activity of mTORC1 [21]. This is also in agreement with the increase in the LC3BII/I ratio with the decrease in serum levels in the culture medium of U87 cells, and, in those conditions, being higher than that found in NHAs (Figure 1L).

Following these initial studies on the characterization of U87 cells grown in the standard and low-serum-content medium, all of the subsequent experiments were performed with cells grown in the latter medium, so that nutrient-starvation conditions were mimicked in vitro, and cellular outcomes of SCD1 silencing could be investigated and exploited from a therapeutic perspective.

U87 cells grown in starvation conditions (2% serum) and transfected with siSCD1 presented significantly lower viability as compared to siNT-transfected cells 72 h after transfection (Figure 3B). When the viability of U87 and U373 cells was assessed in the same conditions (2% serum) at different times after cells have been transfected with siSCD1 or siNT, a significant decrease was noticed only after 68 h (Figure 2B), the time-point at which an increased number of disrupted U87 cells (PI-positive) was registered in siSCD1-transfected cells, as compared to siNT-transfected cells (Figure 2C). The same was not observed when GBM cells were grown in the standard culture medium (10% medium, Figure 2A), which is consistent with our previous results [17], demonstrating that SCD1 silencing was only detrimental to U87 cells under serum-reduced conditions. In fact, although the anti-proliferative effect of inhibiting SCD1 has been observed for some types of cancer cells cultured in the standard medium (10% FBS) [32,38,39], other studies, as ours, have shown that the sensitivity to SCD1 inhibition was enhanced when culturing cancer cells under low FBS conditions [17,40,41,42]. Accordingly, it has been reported that cells might preferentially harvest (from the growth medium) a particular lipid that they are able to synthesize if it is available in sufficient amounts [43]. However, in vivo, cancer cells in the core of a compact tumor (e.g., GBM) mass are expected to grow in starving conditions, which can be mimicked in vitro by reducing the source of most biomolecules (i.e., FBS). In such conditions, lipid composition changes would result exclusively from cell metabolism capacity, through synthesis de novo, or recycling of pre-existent lipids mobilized from LDs. Interestingly, U87 cells grown in FBS-deficient medium seemed to be capable of resorting to autophagy, even after SCD1 downregulation, as deduced from the increase in the LCB3II/LCB3I ratio in siSCD1-transfected cells as compared to control (siNT)-transfected cells (Figure 3A). Furthermore, the significant enhancement of the inhibitory effect of SCD1 silencing on U87 cell viability, induced by a variety of autophagy inhibitors (Figure 3B), emerges as a clear manifestation of the importance of cellular material recycling for the survival of those cells in a nutrient-poor medium.

A central aim of the present work was to identify the alterations in the phospholipid composition of the human GBM U87 cells promoted by SCD1 silencing, in order to highlight the contribution of lipid remodeling to the resistance of these cells to adverse conditions. Although SCD1 has been considered as a promising target for the treatment of a broad spectrum of human malignancies [7,40], as far as we are aware, this is the first time that a phospholipidomic analysis has been applied in GBM cells after SCD1 silencing. Since the role of SCD1 is to catalyze the synthesis of the MUFAs palmitoleic acid and oleic acid from their SFA precursors (Figure S1), the most studied effects of targeting SCD1 activity, either by using pharmacological inhibitors or genetic tools (shRNAs or siRNAs) to downregulate its expression, have been on the fatty acid composition [7]. To the best of our knowledge, there is only one report evaluating the phospholipid molecular species following inhibition of SCD1, which was performed in a human prostate cancer cell line [40].

As a first approach, an analysis of fatty acid relative contents in lipid extracts from cells grown in different conditions was performed (Table 1). It is important to note the absence of effects promoted by the transfection of U87 cells with the control siRNA (siNT), as compared to untreated cells, indicating the specificity of the effects resulting from SCD1 downregulation by siSCD1. By estimating the activities of SCD1 and other key enzymes of fatty acid synthesis on the basis of product-to-substrate ratio calculations, it was noticed that SCD1 silencing induced a decrease in the enzyme activity, as expected, and an increase in the elongase activities assigned to ELOVL-6 and ELOVL-5/6 (Table 2). Consequently, not only was a significant increase in SFA registered (accompanied by a decrease in MUFAs), due to SCD1 downregulation, but also an increase in longer chain fatty acids, that is, 18:0 (at the expense of 16:0) and 18:1 n7 (at the expense of 16:1 n-7), was also observed. Therefore, it is not surprising that SCD1 silencing led to an increase in the conformational order of PL hydrocarbon chains, as detected through the increase in DPH fluorescence polarization (Figure 5).

Curiously, although the UFA (MUFA+PUFA)/SFA decreased as a consequence of SCD1 downregulation, the levels of PUFAs, per se, showed a significant increase, probably in an attempt to counteract the increased membrane order resulting from the predominance of SFAs over MUFAs, for example at the ER level, promoting lipotoxicity. Such putative PUFA-induced compensation of membrane biophysical properties could explain the relatively modest inhibition of cell proliferation induced by SCD1 silencing. On the other hand, the limited supply of lipids by the low-serum-culture medium suggests that PUFAs might have been provided by LDs in a lipophagy process. It is noteworthy that mammalian cells lack the ability to produce fatty acids of the n3 and n6 series; this is the reason why they need to obtain, from the diet, linoleic acid (18:2) and linolenic acid (18:3), which are precursors of n6 PUFAs (e.g., arachidonic acid, 20:4, and docosapentaenoic acid, 22:5) and n3 PUFAs (e.g., eicosatetraenoic acid, 20:4; eicosapentaenoic acid, 20:5; docosapentaenoic acid, 22:5; and docosahexaenoic acid, 22:6), respectively. Since autophagy was promoted by SCD1 silencing, as evidenced by the observed increase in LC3BII/I ratio (Figure 6A), it could have partially contributed to provide siSCD1-transfected cells with fatty acids and other biomolecules, so that cell viability was only partially inhibited (approximately 20% reduction).

However, in terms of the heterogeneous membrane structure, the changes in the fatty acid profile of each PL class emerge as being much more meaningful than the overall alterations in membrane lipid fatty acid composition, the same assumption being applicable to the stability of the lipid monolayer of LDs. In fact, depending on its fatty acid composition, each PL class reveals a particular behavior in hydrophobic-driven lipid/protein associations (i.e., mono and bilayers), reflected by different lipid/lipid and lipid/protein interactions.

It is remarkable that, although SCD1 downregulation had not modified the relative proportions of PL classes as well as the cholesterol/phospholipid molar ratio in U87 cells, fatty acid composition within each PL class was significantly altered, with a predictable impact on cell functioning, namely regarding mitochondrial bioenergetics and autophagy.

PC species with a total of one or two unsaturations (namely, 14:0/18:1 and/or 16:0/16:1; 16:0/18:2 and/or 16:1/18:1 and 18:1/18:1) were significantly decreased, which was accompanied by an increase in disaturated species (i.e., having only saturated fatty acids, namely 14:0/16:0; 16:0/16:0) and those having a monounsaturated fatty acid but longer chains, i.e., 18:0/18:1. Such fatty acid composition changes in PC molecules might translate into more ordered lipid domains, thus favoring, for example, lipid raft stability [44].

In contrast, the PE phospholipid, which is prone to adopt a cone shape when incorporated into polyunsaturated fatty acids, showed, upon SCD1 silencing, an increase in the species containing a saturated chain (18:0) and a polyunsaturated chain, namely 20:4, 22:5, 22:6 and, in the case of plasmenyl-ethanolamine, 20:5, thus anticipating a high propensity to generate lipid packing defects [45]. Remarkably, all PE species contain at least one unsaturation per lipid molecule, and those that contain fatty acids resulting from SCD1 activity (i.e., 16:1 or 18:1), namely the species 16:1/18:1; 16:0/18:1; 18:1/18:1 and 18:1/22:6, underwent, as expected, a significant decrease upon SCD1 downregulation, with the 18:0/18:1 and the 18:1/20:4 species constituting an exception. The molecular geometry of the unsaturated PE species, roughly described as a cone, fits favorably to membrane regions of high curvature, such as those entailed in membrane fusion phenomena [46]. On the other hand, when incorporated into a flat bilayer, cone-shaped molecules create membrane stresses and modify the bilayer lateral pressure profile, with consequences for lipid–lipid and lipid–protein interactions at the membrane level [45]. This particular behavior makes the unsaturated PE species important participants in several cellular processes, namely those involved in autophagy, where PE constitutes the membrane lipid anchor for the protein LC3. LC3 lipidation and membrane insertion are a crucial step in phagophore expansion and autophagosome generation, which depend on the membrane spontaneous curvature [47]. On the other hand, the expressive content of the ethanolamine plasmalogen, which increased upon SCD1 downregulation, is expected to contribute not only to modify membrane physical properties towards those that favor membrane fusion [48], but also to counteract the oxidative damage of the membrane-abundant polyunsaturated phospholipid species, due to its capacity to halt the progression of lipid peroxidation, acting as an endogenous antioxidant [49].

Regarding the SM species, a significant relative abundance of very asymmetric SM species containing a very long fatty acid chain with 24 carbon atoms, whose molecular geometry fits well with that of cholesterol in lipid rafts [44], was found in both conditions (with and without SCD1 silencing).

It is not surprising that the predominant PI species in GBM contains a stearoyl and an arachidonoyl chain (18:0/20:4), since this combination of acyl chains is the most represented (≥70%) in the PI class in mammalian cells, the reason for such preference being a controversial issue [50]. Other acyl combinations have been, however, found in PI, with cancer cells frequently showing PI species containing shorter chains and chains with different levels of unsaturation, such as those identified in the present work [51]. Although our MS-based analysis focused only on PI, it is known that PI and its derived phosphoinositides (PIP, PIP2, PIP3) share a similar fatty acid composition, which might reflect the rapid conversion of PI into its phosphorylated derivatives. An interesting hypothesis, which needs to be clarified, is that the members of the phosphoinositide family are specifically recognized by enzymes and other proteins involved in signal transduction, not only through their headgroups, but also through their acyl chain composition. In the context of autophagy, it is well known that phosphoinositides play an important role in various steps of the process [52]. In terms of the effect of SCD1 silencing, the most notorious effect was the increase in the prevailing PI species (18:0/20:4), as well as those species combining 18:0 with a polyunsaturated chain (20:3, 22:5); species containing MUFAs resulting from the activity of SCD1 (16:1 and 18:1) underwent a decrease in their abundance, excepting the 18:0/18:1 species that increased with SCD1 downregulation.

Additionally, at the level of the molecular species of CL, the pattern of decrease in MUFAs, accompanied by an increase in PUFAs, after SCD1 silencing, was quite evident. Interestingly, the level of CL with linoleic acid and polyunsaturated chains, and of symmetrical and, therefore, mature CLs [27] increased upon SCD1 downregulation. A possible explanation could reside in the mobilization of PUFAs from LDs to specific domains of mitochondrial membranes of GBM cells, favoring CL remodeling through the action of tafazine, whose activity was proposed to be enhanced in high-curvature membrane domains enriched in linoleic acid-containing phospholipids [53].

CL plays a crucial role in the quality control of mitochondria, which increasingly appears to be a pressing need for cancer cells, despite the fact that their energy metabolism is of the glycolytic type [27]. Two processes contribute to the quality of mitochondria, with CL being strongly involved in both: the phenomena of mitochondrial dynamics (fusion and fission) and mitophagy [27,54]. Non-functional or damaged mitochondria are released by fission and recruited into mitophagy. These processes have been shown to be responsible for strengthening the aggressiveness of cancer cells under stress conditions. This could explain the relatively high respiratory reserve capacity of mitochondria from U87 cells grown in medium with low serum concentration. In contrast to fission, which contributes to the quality of the mitochondria, allowing the elimination of obsolete mitochondria, mitochondrial fusion helps to mitigate stress by promoting content mixing of partially damaged mitochondria [54]. After SCD1 silencing, the increase in the proportions of polyunsaturated CLs (presumably with a geometry compatible with a negative membrane curvature) may have contributed to favoring mitochondrial fusion, explaining the existence of relatively functional mitochondria (energetically associated, as reflected by the coupling efficiency parameter), although deprived of the ability to face stressful situations (reflected in the decrease in respiratory reserve capacity). This is an interesting issue that we plan to exploit in the near future, in the context of a combined therapy. In fact, the increase in PUFAs in CL molecular species, in SCD1-silenced cells, should favor high levels of CL oxidation in the presence of a second insult generator of reactive oxygen species (ROS), and, consequently, lead to apoptosis [27]. Thus, the therapeutic association of SCD1 downregulation to new-generation chemotherapeutics, which were shown to be potential inducers of oxidative stress, emerges as a challenging opportunity to efficiently fight the aggressivity of GB cells and counteract their chemoresistance. However, for a therapeutic intervention to be successful, it is mandatory to ensure that it does not adversely affect healthy cells. In this context, previous studies carried out in our laboratory [17] clearly showed that Lipofectamine RNAiMAX/siSCD1 complexes do not promote toxicity in NHAs, although SCD1 mRNA levels were efficiently reduced, by ca. 60%, with respect to control transfection with siNT.

## 4. Materials and Methods

### 4.1. Cells and Reagents

U87 and U373 human glioblastoma (GBM) cells, kindly donated by Dr. Peter Canoll (Columbia University, USA) and Dr. Ernst Wagner (Ludwig-Maximilians University, Germany), respectively, were maintained in Dulbecco Modified Eagle Medium-high glucose (DMEM-HG, D5648) supplemented with 10 mM Hepes, 12 mM NaHCO_3_, 10% (*v*/*v*) heat-inactivated fetal bovine serum (FBS; Thermo Fisher Scientific, Waltham, MA, USA), 100 U/mL penicillin and 100 µg/mL streptomycin. Normal human astrocytes (NHAs) were generously donated by Dr Anne Régnier-Vigouroux (University of Mainz, Germany). NHAs were maintained in DMEM-HG supplemented with 10 mM NaHCO_3_, 2% (*v*/*v*) heat-inactivated FBS, 1% N-2 (Thermo Fisher Scientific), 1% non-essential amino acids, 100 U/mL penicillin and 100 µg/mL streptomycin. Cells were plated 24 h before any experiment at densities of 1.85 × 10^4^ cells/cm^2^ for U87 and 1.55 × 10^4^ cells/cm^2^ for NHA. All cells were cultured at 37 °C under a humidified atmosphere containing 5% CO_2_.

The siRNA duplexes targeting stearoyl-CoA desaturase-1 (siSCD1; sense: 5′-GAGAUAAGUUGGAGACGAUdTdT-3′) and a non-targeting siRNA duplex (siNT; sense 5′-UUCUCCGAACGUGUCACGUdTdT-3′) were synthesized by GeneCust (Ellange, Luxembourg). 3-Methyladenine (3-MA) and wortmannin (WM) were obtained from Selleckchem (Munich, Germany), and stock solutions were prepared in DMSO (Sigma, St. Louis, MO, USA) and stored at −20 °C. Stock solution of chloroquine (CQ diphosphate salt, Sigma) was freshly prepared in sterile water. Oligomycin A, rotenone and antimycin A were obtained from Santa Cruz Biotechnology (Dallas, TX, USA), and stock solutions were prepared in DMSO and stored at −20 °C. Antibodies for SCD1 (stearoyl-CoA desaturase 1; #2438), ACC (acetyl-CoA carboxylase; #3676), pACC (phospho-acetyl-CoA carboxylase; #11818), AMPK (AMP-activated protein kinase; #2603), PI3K (phosphoinositide 3-kinase; #4249), AKT (protein kinase B; #4685), pAKT (phospho-protein kinase B; #4058), HK-II (hexokinase II; #2867), LC3B (microtubule-associated protein 1 light chain 3 isoform B; #2775) and α-tubulin (#3873) were purchased from Cell Signaling Technology (Leiden, The Netherlands). Antibody for β-actin (A5441) was purchased from Sigma. The secondary antibodies anti-rabbit IgG-AP (NIF1317) and anti-mouse IgG + IgM-AP (NIF1316) were obtained from GE Healthcare (Little Chalfont, UK). All other reagents were obtained from Sigma unless stated otherwise.

### 4.2. Cell Transfection and Addition of Autophagy Inhibitors

Complexes of siRNA with Lipofectamine RNAiMAX (Life Technologies, Carlsbad, CA, USA) were prepared as described previously [17]. Twenty-four hours after plating, the cells were incubated with the siRNA/Lipofectamine RNAiMAX complexes at 50 nM in complete culture medium supplemented with 2 or 10% FBS at 37 °C for 4 h. After the incubation period, the transfection medium was removed, and fresh medium containing 2 or 10% FBS was added to the cells. The time after transfection with siRNA began after removal of the complexes.

CQ (25 µM), WM (5 µM) or 3-MA (5 mM) were added to U87 cells 20 h after siRNA transfection in medium containing 2% FBS followed by 48 h of incubation at 37 °C.

### 4.3. Cell Proliferation, Viability and Death Analyses

Cell proliferation was measured by the sulforhodamine B (SRB) colorimetric assay, as described previously [18,55]. U87 cells were seeded onto 96-well plates in growth medium containing 2% or 10% FBS. Cells were fixed with trichloroacetic acid (final concentration 10% *w*/*v*, 4 °C, overnight) at several timepoints for at least 5 days. Each well was washed with water, dried, stained using 200 µL SRB solution (0.057% *w*/*v* in 1% *v*/*v* acetic acid) for 1 h at 37 °C, washed with 1% (*v*/*v*) acetic acid, and dried. Cell-bound SRB was solubilized with 200 µL of Tris buffer (10 mM, pH 10), and the absorbance was measured at 510 nm in a microplate reader (SPECTRAmax PLUS 384, Molecular Devices, Sunnyvale, CA, USA). The doubling time was calculated using the GraphPad Prism exponential growth curve equation.

Cell viability was assessed by a modified Alamar Blue assay, using resazurin dye in growth medium, as described previously [18]. The absorbance of the medium at 570 and 600 nm was measured in a microplate reader (SPECTRAmax PLUS 384), and the cell viability was calculated as a percentage of non-treated cells, according to Equation (1):

(1) $$\mathrm{Cell}\;\mathrm{viability}\;(\%\;\mathrm{of}\;\mathrm{NTC})=\left[\left(A_{570}-A_{600}\right)/\left({A^{\prime }}_{570}-{A^{\prime }}_{600}\right)\right]\times 100\%$$

where *A*_570_ and *A*_600_ are the absorbances of the samples and ${A^{\prime }}_{570}$ and ${A^{\prime }}_{600}$ are those of the control (NTCs, non-treated cells), at the indicated wavelengths.

For total cell death assessment, U87 cells were collected and washed by centrifugation (300× *g*, 4 °C, 5 min) in ice-cold phosphate-buffered saline (PBS; 137 mM NaCl, 2.7 mM KCl, 8.1 mM Na_2_HPO_4_, 1.5 mM KH_2_PO_4_, pH 7.3). Cells were resuspended in 100 µL of PBS, and 1 µL propidium iodide (PI) solution (50 µg/mL in PBS) was added to cells. After 15 min incubation in the dark at room temperature, 200 µL of PBS were added to each sample, and cells were immediately analyzed in a Becton Dickinson FACSCalibur flow cytometer (BD Biosciences, San Jose, CA, USA) using a 488 nm argon excitation laser, and the emitted PI fluorescence was measured on FL3 with a 670 nm longpass filter. A minimum of 10,000 events were collected for each sample. PI positive staining cells were considered as dead cells.

### 4.4. Quantitative Real-Time PCR (qRT-PCR)

Total RNA was extracted from cells using the NucleoSpin RNA Isolation Kit (Macherey-Nagel, Düren, Germany) and quantified using a NanoDrop 2000 (Thermo Fisher Scientific). For each sample, 500 ng of total RNA were converted into cDNA in the thermal cycler VWR Unocycler (VWR, Radnor, PA, USA) using the NZY First-Strand cDNA Synthesis Kit (NZYTech, Lisbon, Portugal). qRT-PCR was performed in a StepOnePlus Real-Time PCR System (Applied Biosystems, Foster City, CA, USA) using diluted cDNA (1:20 in RNase-free water) and the SsoAdvanced Universal SYBR Green Supermix (Bio-Rad, Hercules, CA, USA). The reaction conditions were previously described [56]. The primers for the target gene (SCD1) and the reference gene (HPRT1) were pre-designed by Qiagen (QuantiTect Primer, Qiagen, Hilden, Germany). SCD1 mRNA fold decrease caused by cell treatment with siSCD1, with respect to cells treated with siNT, was determined following the Pfaffl method [57] for relative mRNA quantification, in the presence of target and reference genes with different amplification efficiencies. The amplification efficiency for SCD1 or HPRT1 gene was determined according to the formula: *E =* 10^(−1/*S*)^, where *S* is the slope of the standard curve obtained for each gene.

### 4.5. Western Blot Analysis

Cells were lysed using lysis buffer containing 20 mM Tris-HCl (pH 7.5) 0.15 M NaCl, 1 mM Na_2_EDTA, 1 mM EGTA, 0.1% SDS, 1% NP40, 1% sodium deoxycholate, supplemented with a protease inhibitor cocktail (SigmaFAST Protease Inhibitor Cocktail Tablets, EDTA-Free), 0.5 mM DTT, 1 mM PMSF, 1 mM NaF and 1 mM Na_3_VO_4_ at 4 °C, followed by three freeze/thaw cycles in liquid nitrogen. Lysates were centrifuged at 16,000× *g* at 4 °C for 15 min, and the total protein content of the supernatants quantified using the Bio-Rad DC protein assay (Bio-Rad). Forty micrograms of total protein were diluted in Laemmli buffer, denatured upon 5 min incubation at 95 °C, and loaded onto a 7.5 or 10% polyacrylamide gel. After electrophoresis, proteins were blotted onto a PVDF membrane at 750 mA for 2 h (Millipore, Merck KGaA, Darmstadt, Germany) and blocked with 5% (*w*/*v*) non-fat milk in Tris-buffered saline with Tween-20 (TBST; 20 mM Tris–HCl, pH 7.6, 137 mM NaCl, 0.1% Tween) at room temperature for 1 h. Membranes were incubated overnight at 4 °C with rabbit anti-SCD1 (1:1000), anti-ACC (1:1000), anti-pACC (1:1000), anti-AMPK (1:1000), anti-PI3K (1:1000), anti-AKT (1:1000), anti-pAKT (1:1000), anti-HK-II (1:1000) or anti-LC3B (1:1000) primary antibodies. The membrane was then washed thrice with TBST (10 min each) and incubated with anti-rabbit alkaline phosphatase-labeled secondary antibody (1:10,000) at 4 °C for 2 h. After incubation with ECF substrate (GE Healthcare) at room temperature for a maximum of 5 min, protein bands were detected using a ChemiDoc Touch Imaging System (Bio-Rad). As an internal control for protein loading, the membrane was re-probed with mouse anti-α-tubulin (1:1000) or mouse anti-β-actin (1:5000) antibody and, thereafter, with anti-mouse alkaline phosphatase-labeled secondary antibody (1:10,000). Band intensities were obtained using the Quantity One software (Bio-Rad) and normalized to the corresponding α-tubulin or β-actin levels for each condition.

### 4.6. Lipid Extraction and Quantification of Total Phospholipid and Cholesterol

Cell pellets were resuspended in 1 mL of ice-cold water and sonicated. Aliquots (4 μL) were taken for protein quantification using the Bio-Rad DC protein assay (Bio-Rad). Then, total lipids were extracted from cells using the Bligh and Dyer procedure, as described previously [18].

Aliquots of lipid extract (50 μL) were placed into glass tubes and allowed to air dry. The total amount of phospholipid was quantified using a phosphorus assay, according to Rouser et al. [58] with some modifications. Briefly, 0.163 mL of 70% (*v*/*v*) perchloric acid was added to the dried lipids, followed by incubation at 180 °C for 2 h in a heat block, with a glass marble covering each tube. Then, 0.825 mL water, 0.125 mL of 2.5% (*w*/*v*) ammonium molybdate, and 0.125 mL of 10% (*w*/*v*) ascorbic acid (freshly prepared) were added to each tube, followed by incubation for 10 min in a boiling water bath, and the absorbance of each sample was then measured in a microplate reader (SPECTRAmax PLUS 384) at 800 nm. The amount of phosphorus was determined from a standard curve obtained by using aliquots of a Phosphorus Standard Solution (Sigma), containing 3.25 to 156 nmol of phosphorus, which were submitted to the same treatment as the samples.

Total cholesterol was quantified using the Amplex Red Cholesterol Assay Kit (Thermo Fisher Scientific). Briefly, aliquots of lipid extract (5 μL) were allowed to air dry and then suspended in the reaction buffer (160 μL) from the kit. Reactions took place in a black 96-well plate by the addition of 50 μL of Amplex Red working solution with 50 μL of assay sample, according to the manufacturer’s instructions. Fluorescence was measured in a fluorescence microplate reader (SPECTRAmax Gemini EM fluorimeter Molecular Devices, Sunnyvale, CA, USA) at excitation/emission of 560/590 nm.

### 4.7. Analysis of Fatty Acid Profiles by Gas Chromatography (GC)-Mass Spectrometry (MS)

Fatty acid profiling was evaluated by the analysis of fatty acid methyl esters (FAMEs) obtained by transesterification [59]. Briefly, 1 mL of hexane was added to 15 μg of dried phospholipid extract. FAMEs were obtained by sequentially adding 200 μL of 2 M KOH in methanol and 2 mL of saturated NaCl solution, followed by intense vortexing in between. After centrifugation, the organic phase was collected and dried under a nitrogen stream. The resulting FAMEs were dissolved in hexane prior to injection and analyzed in an Agilent Technologies 6890N Network gas chromatograph (Santa Clara, CA, USA) equipped with a DB-FFAP column with 30 m of length, 0.32 mm of internal diameter, and 0.25 µm of film thickness (J&W Scientific, Folsom, CA, USA). The GC was connected to an Agilent 5973 Network Mass Selective Detector operating with an electron impact mode at 70 eV and scanning the range *m*/*z* 50–550 in a 1 s cycle in a full scan mode acquisition. The initial oven temperature was 80 °C, the first ramp rate was 25 °C·min^−1^ to 160 °C, then from 160 to 210 °C at 2 °C·min^−1^, and finally until 250 °C at 30 °C·min^−1^. The injector and detector temperatures were 220 and 250 °C, respectively. Helium was used as carrier gas at a flow rate of 1.3 mL·min^−1^. FAMEs were identified by comparison of spectra with those available in the Wiley275 database and the FAME archive from AOCS Lipid Library. The relative amounts of FAs were calculated by the percent area method, considering the sum of areas of the identified FAs.

### 4.8. Thin-Layer Chromatography (TLC) of Phospholipids (PLs)

Separation of PL classes by TLC from the lipid extract was performed using Whatman LK5 thin-layer plates and according to Vaden et al. [60]. Briefly, the plates were washed in chloroform/methanol (1/1, *v*/*v*) and treated with boric acid in ethanol (1.8% *w*/*v*) and activated at 100 °C for 10 min. Then, the plates were spotted with 40 μg of phospholipid extract, and TLC plates were developed in a mixture of solvents consisting of chloroform/ethanol/water/triethylamine (30/35/7/35, *v*/*v*/*v*/*v*). To reveal the phospholipid spots, TLC plates were sprayed with a primuline solution (5 mg in 100 mL of acetone/water, 80/20, *v*/*v*) and visualized under UV light (λ = 340 nm). PLs were identified taking into consideration the distances of the spots from the point of origin and the front of the solvent (retention factor, Rf, values), as compared to those of the spots of standard PLs run in the same plate. Two spots per sample were scraped off from the plates directly into tubes and quantified using the phosphorus assay, as described above. Prior to spectrophotometric determination, samples were centrifuged for 5 min at 500× *g* to promote silica sedimentation. The percentage of PL class was calculated relative to the total amount of phosphorus in the sample.

### 4.9. Analysis of PL Molecular Species by Liquid Chromatography (LC)-MS

To perform LC-MS analysis of lipid extracts, a high-performance LC (HPLC) system (Thermo scientific Accela) coupled online to a linear ion trap (LXQ; Thermo Finnigan, San Jose, CA, USA) mass spectrometer was employed. The solvent system consisted of two mobile phases, as follows: mobile phase A (acetonitrile:methanol:water 55:35:10 (*v*/*v*) with 10 mM ammonium acetate) and mobile phase B (acetonitrile:methanol 60:40 (*v*/*v*) with 10 mM ammonium acetate). Initially, 0% of mobile phase A was held isocratically for 10 min, followed by a linear increase to 100% of A within 10 min and a maintenance period of 25 min, returning to the initial conditions in 10 min. Aliquots of 25 μg of total phospholipid were diluted in 90 μL of mobile phase B, filtered, and 10 μL was introduced into the Ascentis Si column (15 cm × 1 mm, 3 µm) with a flow rate of 40 µL min^−1^ and at 30 °C. The LXQ was operated in both positive (electrospray voltage +5 kV) and negative (electrospray voltage −4.7 kV) modes at 275 °C capillary temperature with a sheath gas flow of 8 U. Normalized collision energy (CE) was 27 (arbitrary units) for MS/MS. Data acquisition was carried out on an Xcalibur™ data system (V2.0, Thermo Fisher Scientific). Relative quantification of individual phospholipid species was achieved by determination of the ratio between the area of reconstructed ion chromatogram of a given *m*/*z* value against the sum of the reconstructed areas considered for each class.

### 4.10. Fluorescence Polarization Assay

Lipids extracts from U87 cells, either non-treated or transfected with siSCD1 and with siNT, grown in medium containing 2% FBS, were used to prepare liposomes (multilamellar vesicles) according to a protocol described previously [61], to which a small amount of a 2 mM 1,6-diphenyl-1,3,5-hexatriene (DPH) solution in tetrahydrofuran was added under vortex. Liposome preparation and probe addition were performed at 55 °C, a temperature that was predictably above the phase transition temperature of all of the lipid mixtures, which were then incubated at room temperature in the dark for a period of 15 h. Fluorescence measurements in the DPH-labeled liposomes, performed in a Perkin Elmer LS 55B fluorescence spectrophotometer (Perkin Elmer, Waltham, MA, USA) equipped with polarization filters at the temperature range from 10 to 50 °C were corrected for the contribution of light scattering by using appropriate blanks without added probe and with equivalent volumes of tetrahydrofuran. The excitation wavelength was set at 336 nm, and the emission wavelength at 450 nm (5 nm excitation and 6 nm emission band pass). The fluorescence polarization (P) was calculated according to Shinitzky and Barenholz [62], from the equation

P = I_ΙΙ_ − G I_⊥_/I_ΙΙ_ + G I_⊥_ (2)

where I_II_ and I_⊥_ are the intensities of the light emitted with its polarization plane parallel and perpendicular to that of the exciting beam, respectively. G, the instrumental grating factor, is given by the ratio of vertically to horizontally polarized emission components when the excitation light is polarized in the horizontal plane.

### 4.11. Oxygen Consumption Assay

Real-time oxygen consumption rates (OCRs) were determined using the Seahorse XFe24 extracellular flux analyzer (Agilent, Santa Clara, CA, USA). Briefly, 96 h after plating the cells in XFe24 microplates, cells were washed in pre-warmed assay medium (D5030 supplemented with 31.6 mM NaCl, 25 mM glucose, 4 mM L-glutamine, and adjusted to 7.35 ± 0.05 pH). Cells were then maintained in 450 μL/well of assay medium at 37 °C in a non-CO_2_ incubator for 1 h. During the incubation time, 50 μL of 10 μM oligomycin, 55 μL of 10.1 μM FCCP, and 60 μL of 10.3 μM rotenone/10.3 μM antimycin A, prepared in assay medium, were sequentially loaded into the injection ports in the XFe24 sensor cartridge. Four OCR measurement cycles were performed before the addition of inhibitors (baseline OCR) and after the sequential addition of oligomycin (to inhibit ATP synthase), FCCP (to disrupt the mitochondrial membrane potential), and a mix of rotenone and antimycin A (to inhibit complexes I and III, respectively). Each OCR measurement cycle consisted of 2 min of mixing, 30 s of waiting, and 4 min of OCR recording. Mitochondrial respiration parameters, namely non-mitochondrial respiration (OCR after rotenone/antimycin A injection), mitochondrial basal respiration (baseline OCR minus rotenone/antimycin A response), ATP-linked respiration (baseline OCR minus oligomycin response), proton leak (oligomycin response minus rotenone/antimycin A response), maximal respiration (FCCP response minus rotenone/antimycin A response), spare respiratory capacity (maximum OCR minus baseline OCR) and coupling efficiency (mitochondrial basal respiration divided by ATP-linked respiration), were determined after OCR measurement normalization in terms of the protein content in each well, evaluated by the SRB assay.

### 4.12. Statistical Analysis

Results from all experiments were represented as mean ± SD. The value “n” refers to the number of independent experiments. Data were analyzed using GraphPad Prism 6 Software (La Jolla, CA, USA). Statistical significance was set at *p* < 0.05.

## Figures and Tables

**Table 1:** Relative abundance (%) of fatty acids (FAs) in U87 cell lipids. Cells, grown in medium containing 2% FBS were transfected with siRNA against SCD1 (siSCD1) or a non-targeting siRNA sequence (siNT) complexed with Lipofectamine RNAiMAX. Sixty-eight hours after transfection, lipids were extracted and their FA composition determined by GC-MS.

FA ^a^	NTC ^b^	siNT	siSCD1
Saturated FA (SFA)			
14:0	1.10 ± 0.18	1.16 ± 0.15	1.11 ± 0.06
16:0	19.14 ± 2.27	18.63 ± 1.77	19.01 ± 0.84
18:0	14.2 ± 1.88 ***	14.74 ± 0.90 **	21.42 ± 0.72
20:0	0.42 ± 0.05 **	0.42 ± 0.01 **	0.67 ± 0.08
24:0	0.75 ± 0.05	0.78 ± 0.33	0.96 ± 0.08
Monounsaturated FA (MUFA)			
16:1 n-9	0.73 ± 0.06	0.70 ± 0.13	0.82 ± 0.07
16:1 n-7	5.94 ± 0.72 ***	5.17 ± 0.19 ***	1.79 ± 0.20
18:1 n-9	24.59 ± 0.75 **	25.15 ± 0.85 **	20.84 ± 0.90
18:1 n-7	10.42 ± 0.66 ***	10.03 ± 0.46 ***	6.69 ± 0.43
18:1	0.63 ± 0.05	0.63 ± 0.11	0.49 ± 0.07
20:1	1.53 ± 0.17 *	1.61 ± 0.08 **	1.11 ± 0.10
20:1	0.88 ± 0.10 *	0.78 ± 0.07	0.58 ± 0.12
Polyunsaturated FA (PUFA)			
18:2 n-6	2.09 ± 0.08 ***	2.15 ± 0.12 ***	2.73 ± 0.04
20:2	0.38 ± 0.03	0.40 ± 0.02	0.45 ± 0.06
20:3	0.40 ± 0.07	0.36 ± 0.07	0.43 ± 0.07
20:3 n-6	0.82 ± 0.14 **	0.77 ± 0.05 **	1.22 ± 0.05
20:4 n-6	4.94 ± 0.67 *	4.96 ± 0.32 *	6.27 ± 0.06
22:4 n-6	1.57 ± 0.24	1.81 ± 0.19	1.78 ± 0.15
22:5 n-3	4.02 ± 0.56	4.42 ±0.77	5.14 ± 0.20
22:6 n-3	5.42 ± 0.54	5.34 ± 0.98	6.49 ± 0.55
Sum			
SFA	35.61 ± 3.40 *	35.73 ± 2.24 *	43.16 ± 1.45
MUFA	44.72 ± 2.11 ***	44.07 ± 0.39 ***	32.32 ± 1.16
PUFA	19.66 ± 2.29 *	20.20 ± 1.96 *	24.51 ± 0.59
n-6	9.43 ± 1.11 **	9.69 ± 0.33 *	12.00 ± 0.15
n-3	9.45 ± 1.08	9.75 ± 1.74	11.64 ± 0.68
UFA ^c^	64.39 ± 3.40 *	64.27 ± 2.24 *	56.84 ± 1.45
Ratio			
MUFA/SFA	1.27 ± 0.18 **	1.24 ± 0.09 **	0.75 ± 0.05
PUFA/SFA	0.56 ± 0.12	0.57 ± 0.09	0.57 ± 0.03
UFA/SFA	1.83 ± 0.28 *	1.81 ± 0.18 *	1.32 ± 0.08
n-6/n-3 PUFA	1.00 ± 0.03	1.01 ± 0.14	1.03 ± 0.06

^a^ The relative amounts of FAs were calculated by the percent area method, considering the sum of the areas of the identified FAs as 100% (*n* = 3; one-way ANOVA followed by Dunnett’s test; * *p* < 0.05, ** *p* < 0.01, *** *p* < 0.001 vs. siSCD1); ^b^ non-treated cells; ^c^ unsaturated FAs (MUFAs + PUFAs).

**Table 2:** Values of indexes for fatty acid desaturation and elongation activities of different enzymes.

Name	Index ^a^	NTC	siNT	siSCD1
SCD1-16	16:1 n-7/16:0	0.31 ± 0.04 ***	0.28 ± 0.02 ***	0.09 ± 0.01
SCD1-18	18:1 n-9/18:0	1.75 ± 0.27 **	1.71 ± 0.17 **	0.97 ± 0.07
ELOVL-6	18:0/16:0	0.75 ± 0.10 **	0.79 ± 0.05 **	1.13 ± 0.04
ELOVL-6 + SCD1-18	18:0 + 18:1 n-9/16:0	2.05 ± 0.25	2.15 ± 0.20	2.23 ± 0.13
ELOVL-1/3/7	20:0/18:0	0.030 ± 0.005	0.028 ± 0.002	0.031 ± 0.004
ELOVL-5/6	18:1 n-7/16:1 n-7	1.76 ± 0.14 ***	1.94 ± 0.07 ***	3.75 ± 0.23
SCD1-16 + ELOVL-5/6	16:1 n-7 + 18:1 n-7/16:0	0.86 ± 0.13 **	0.82 ± 0.07 **	0.45 ± 0.04

^a^ Ratios were calculated from relative FA percentages presented in Table 1 (*n* = 3; one-way ANOVA followed by Dunnett’s test; ** *p* < 0.01, *** *p* < 0.001 vs. siSCD1).

## Data Availability

Not applicable.

## Supplementary Materials

The following supporting information can be downloaded at: https://www.mdpi.com/article/10.3390/ijms232113014/s1, Figure S1: Biosynthesis pathways of the identified SFA and MUFA species.; Table S1: MS- and MS/MS-based identification of the phospholipid (PL) molecular species that were quantified in the present study.
