# Supplementary material for: Stearoyl CoA Desaturase-1 Silencing in Glioblastoma Cells: Phospholipid Remodeling and Cytotoxicity Enhanced upon Autophagy Inhibition
Source: Int J Mol Sci. 2022 Oct 27;23(21):13014. doi: 10.3390/ijms232113014 (PMC9654881; doi:10.3390/ijms232113014)
Supplement: Supplementary file 1 [file ijms-23-13014-s001.zip › ijms-1919074-supplementary.pdf]

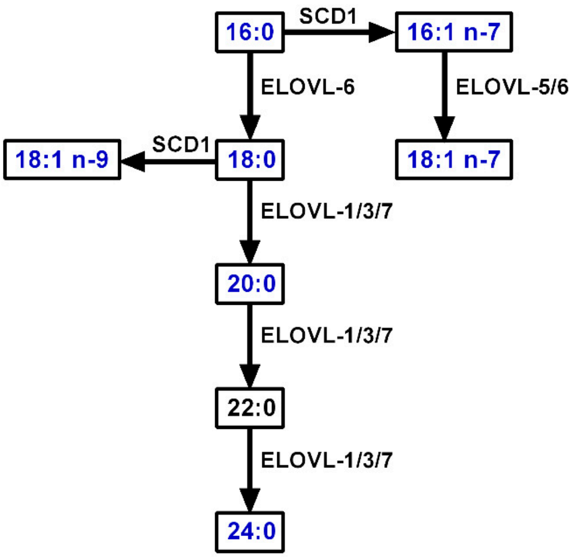

Figure S1. Biosynthesis pathways of the identified SFA and MUFA species (in blue).

Table S1- MS- and MS/MS-based identification of the phospholipid (PL) molecular species that were quantified in the present study. Fatty acyl chain composition was deduced from the carboxylate ions observed in negative mode. In the cases in which a positive charge ion was chosen for the detection, the negative ion counterpart was checked to assign the fatty acyl chain composition. Exceptionally, sphingomyelins fatty acyl composition was observed in positive mode based on the sphingoid base ions. Data are presented as *m/z* values (ratios of mass to charge) and using a shorthand notation (C:N, indicating the number of carbon atoms, C, and double bonds, N) for each fatty acid of the molecular species. The most abundant molecular species of each PL class present in U87 cells grown in low serum-containing medium are shown in blue. The PL molecular species with a relative abundance above 10% are indicated in bold. The direction of the alterations observed in PL molecular species resulting from SCD1 silencing (siSCD1) as compared with control transfection (siNT) are generally referred as Up or Down in the last column.

Class	Molecular species C:N	Fatty acids	Ion	Observed *m/z*	Error (ppm)	Effect of SCD1 silencing (siSCD1 vs. siNT)
PC	30:0	14:0/16:0	$[M + H]^+$	706.5371	-1.4	Up
	**32:1**	**14:0/18:1; 16:0/16:1**	**$[M + H]^+$**	**732.5525**	**-1.8**	**Down**
	32:0	16:0/16:0	$[M + H]^+$	734.5672	-3.0	Up
	**34:2**	**16:0/18:2; 16:1/18:1**	**$[M + H]^+$**	**758.5684**	**-1.3**	**Down**
	**34:1**	**16:0/18:1**	**$[M + H]^+$**	**760.5838**	**-1.7**	
	36:4	16:0/20:4	$[M + H]^+$	782.5658	-4.6	
	36:3	16:0/20:3; 18:1/18:2	$[M + H]^+$	784.5829	-2.8	
	**36:2**	**18:1/18:1**	**$[M + H]^+$**	**786.5992**	**-1.9**	**Down**
	36:1	18:0/18:1	$[M + H]^+$	788.6123	-5.2	Up
	38:5	16:0/22:5; 18:1/20:4	$[M + H]^+$	808.5817	-4.2	
PE	34:2	16:1/18:1	$[M - H]^-$	714.5071	-1.1	Down
	**34:1**	**16:0/18:1**	**$[M - H]^-$**	**716.5225**	**-1.5**	**Down**
	**36:2**	**18:1/18:1**	**$[M - H]^-$**	**742.5381**	**-1.5**	**Down**
	**36:1**	**18:0/18:1**	**$[M - H]^-$**	**744.5530**	**-2.6**	**Up**
	P-38:5	P-18:0/20:5	$[M - H]^-$	748.5269	-2.4	Up
	**38:5**	**18:1/20:4**	**$[M - H]^-$**	**764.5220**	**-2.1**	
	**38:4**	**18:0/20:4**	**$[M - H]^-$**	**766.5374**	**-2.3**	**Up**
	40:7	18:1/22:6	$[M - H]^-$	788.5219	-2.2	Down
	40:6	18:0/22:6	$[M - H]^-$	790.5376	-2.0	Up

	40:5	18:0/22:5	$[M - H]^-$	792.5525	-3.0	Up
SM	**d34:1**	**d18:0/16:1; d18:1/16:0**	**$[M + H]^+$**	**703.5738**	**-1.4**	
	d40:1	d18:0/22:1; d18:1/22:0	$[M + H]^+$	787.6676	-1.4	
	**d42:2**	**d18:1/24:1**	**$[M + H]^+$**	**813.683**	**-1.7**	**Down**
	**d42:1**	**d18:0/24:1; d18:1/24:0**	**$[M + H]^+$**	**815.6977**	**-2.8**	**Up**
PI	34:2	16:0/18:2; 16:1/18:1	$[M - H]^-$	833.5175	-1.3	Down
	34:1	16:0/18:1	$[M - H]^-$	835.5328	-1.7	
	36:4	16:0/20:4	$[M - H]^-$	857.5165	-2.4	
	36:3	16:0/20:3	$[M - H]^-$	859.532	-2.6	
	**36:2**	**18:1/18:1**	**$[M - H]^-$**	**861.5486**	**-1.5**	**Down**
	36:1	18:0/18:1	$[M - H]^-$	863.5623	-3.7	Up
	**38:5**	**18:1/20:4**	**$[M - H]^-$**	**883.5324**	**-2.0**	**Down**
	**38:4**	**18:0/20:4**	**$[M - H]^-$**	**885.5487**	**-1.4**	**Up**
	**38.3**	**18:0/20:3**	**$[M - H]^-$**	**887.5625**	**-3.4**	**Up**
	40:6	18:0/22:6; 18:1/22:5	$[M - H]^-$	909.5483	-1.8	
	40:5	18:0/22:5	$[M - H]^-$	911.5631	-2.6	Up
CL	66:4	-	$[M - H]^-$	1371.9279	-4.2	
	66:3	-	$[M - H]^-$	1373.9401	-6.7	
	68:5	-	$[M - H]^-$	1397.9432	-4.1	Down
	**68:4**	**-**	**$[M - H]^-$**	**1399.9600**	**-3.5**	**Down**
	**68:3**	**-**	**$[M - H]^-$**	**1401.9718**	**-6.3**	**Down**
	70:6	-	$[M - H]^-$	1423.9576	-5.1	
	**70:5**	**-**	**$[M - H]^-$**	**1425.9756**	**-3.5**	
	**70:4**	**-**	**$[M - H]^-$**	**1427.9896**	**-4.6**	
	72:7	-	$[M - H]^-$	1449.9727	-5.4	
	**72:6**	**-**	**$[M - H]^-$**	**1451.9881**	**-5.6**	**Up**
	74:8	-	$[M - H]^-$	1475.9884	-5.3	Up
